# Continuous epidural anesthesia with double catheters for cesarean section in a patient with severe pulmonary hypertension: A case report

**DOI:** 10.1097/MD.0000000000027979

**Published:** 2021-11-24

**Authors:** Pingzhu Wang, Xiaojing Chen, Jingwen Zhang, Yushan Ma

**Affiliations:** a Department of Anesthesiology, West China Second University Hospital, Sichuan University, Chengdu, Sichuan, PR China; b Key Laboratory of Birth Defects and Related Women and Children Diseases, Ministry of Education, Chengdu, Sichuan, PR China.

**Keywords:** anaesthetic management, case report, continuous epidural anesthesia with double catheters, pregnant woman, pulmonary hypertension

## Abstract

**Rationale:**

Pregnancy in a woman with pulmonary hypertension (PH) carries prohibitively high risks of cardiopulmonary complications and high maternal and fetal morbidity and mortality. Anaesthetic management during delivery or cesarean section is very important for the prognosis of pregnant women with PH. The choice between general anesthesia or intraspinal anesthesia is controversial. There have been few case reports of anesthetic management under continuous epidural anesthesia with double catheters in such patients.

**Patient concerns:**

A 35-year-old pregnant woman presented to the emergency department with fatigue and shortness of breath for 10 days at 16 weeks of gestation.

**Diagnosis:**

According to transthoracic echocardiogram, her pulmonary artery pressure (PAP) was 75 mm Hg, and she had a dilated left ventricle (67 mm) and a ventricular septal defect (1.7 mm) with a bidirectional shunt.

**Interventions:**

Elective cesarean section under continuous epidural anesthesia with double catheters to terminate a pregnancy in order to avoid development of cardiac failure.

**Outcomes:**

The pregnant woman underwent cesarean section safely and steadily under continuous epidural anesthesia with double catheters. She was discharged on the seventh postoperative day.

**Lessons:**

The advantages of continuous epidural anesthesia with double catheters are stable hemodynamics and complete analgesia. The continuous epidural anesthesia with double catheters can be applied to patients with cardiopulmonary disease like severe PH. Compared with general anesthesia, spinal anesthesia, and single-catheter epidural anesthesia continuous epidural anesthesia is a better option for patients with both PH and heart failure.

## 1 Introduction

Pregnancy with pulmonary hypertension (PH) carries prohibitively high risks of cardiopulmonary complications and high maternal and fetal morbidity and mortality. The maternal mortality of pregnancy with severe pulmonary hypertension is reportedly 5% to 25%, and neonatal mortality is reported to be as high as 18%.^[1]^ Therefore, PH patients are contraindicated to become pregnant in practice guidelines or terminate the pregnancy in the first trimester^[2,3]^; however, only 60% of PH patients are diagnosed before pregnancy, and 30% are diagnosed during pregnancy.^[4]^ In pregnancy with PH, exacerbation of PH and heart failure usually occur during 20 to 24 weeks of gestation, during childbirth or within 1 month after delivery. Therefore, anesthetic management during delivery or cesarean section is very important for the prognosis of pregnant women with PH.^[5]^ The choice between general anesthesia or intraspinal anesthesia is controversial. There have been few case reports of anesthetic management under continuous epidural anesthesia with double catheters in such patients.

## 2 Case report

We received written consent from the patient prior to publication of this case.

A 35-year-old multiparous woman with her fifth pregnancy and fourth labor presented with fatigue and shortness of breath for 10 days at 16 weeks of gestation. During the past 13 years, she had delivered vaginally 3 times without any complications. During this pregnancy, she had not been seen by any obstetrician until she had noticed considerable fatigue and dyspnea for 10 days. According to transthoracic echocardiogram, her pulmonary artery pressure (PAP) was 75 mm Hg, and she had a dilated left ventricle (67 mm) and a ventricular septal defect (1.7 mm) with a bidirectional shunt. On examination, she could only sleep in a semi reclining position. Rhythmic cardiac tones with an accentuated pulmonary component of the second heart sound were found on auscultation, with a heart rate of 80 beats per minute, blood pressure of 104/80 mm Hg and oxyhemoglobin saturation of 93% on room air. Electrocardiography showed sinus rhythm, incomplete right bundle branch block, and left anterior branch block.

The patient with severe PH developed right heart failure early in the second trimester and was at a high risk for sudden death. In discussion with her cardiologist, obstetrician and anesthesiologist, based on maternal will, it was decided to chose an artificial abortion by cesarean section under continuous epidural anesthesia with double catheters to avoid development of cardiac failure at 16 weeks and 2 days of gestation.

Invasive arterial blood pressure and central venous pressure (CVP) were administered for hemodynamic monitoring. The patient started with 104/80 mm Hg blood pressure, 80 beats per minute heart rate, 18 mm Hg CVP, and oxyhemoglobin saturation of 97% to 100% with an oxygen mask (6 L/min) before anesthesia. The arterial blood gas analysis showed pH 7.447, PaCO_2_ 27 mm Hg, PaO_2_ 77 mm Hg, SO_2_ 95%, BE −5 mmol/L, HCO_3_^-^ 18.7 mmol/L, and K^+^ 4.0 mmol/L. With the head of the bed elevated 30 degrees, an epidural catheter (the lower catheter) was inserted 3 cm caudad first at the L3–4 interspace, and then another epidural catheter (the upper catheter) was inserted 2 cm cephalad at the L1–2 interspace. The epidural puncture was performed by a skilled staff, and it took 10 minutes in total. Test doses of the 2 epidural catheters both showed negative results. Norepinephrine was continuously pumped to maintain blood pressure, with an initial dose of 0.05 μg·kg^−1^·minutes^−1^. 6 mL of 2% lidocaine were added to the upper catheter after 10 minutes of the test dose, and another 4 mL was added after 15 minutes of observation. Moreover, 6 mL of 1.5% lidocaine was used in the lower catheter 15 minutes after the test dose, and another 4 mL was added 15 minutes after observation. The block level was estimated 10 minutes after each dose by temperature testing, and the operation began immediately upon the block level reached the T4 segmental level. During adjustment of the block level, the infusion speed of norepinephrine was 0.02 to 0.06 μg·kg^−1^·minutes^−1^, blood pressure fluctuated between 100 to 128/60 to 75 mm Hg, and CVP decreased to 12 mm Hg. A fetus was removed 6 minutes after the start of the operation, followed by intravenous infusion of oxytocin at a speed of 2 u/h. The blood pressure gradually decreased to 80/55 mm Hg, so noradrenaline was increased to 0.06 to 0.1 μg·kg^−1^·minutes^−1^ to maintain blood pressure at 89 to 122/50 to 72 mm Hg. During surgery, total blood loss was 600 mL, urine was 200 mL, and crystalloid fluid was infused at 500 mL. Multimodal postoperative analgesia consisting of a self-controlled intravenous analgesia pump (1200 mg tramadol and 180 mL saline) and bilateral ventral transverse fascia block (15 mL of 0.25% ropivacaine on each side) was performed to relieve the pain after surgery.

On the 3rd day after surgery, the PAP was 75 mm Hg, and there was no reduction. The cardiologist recommended that the patient take ambrisentan tablets to expand the pulmonary artery and visit the cardiac surgery outpatient clinic 1 week after surgery. Her condition remained stable, and she returned to the general ward on the 5th day after surgery and was discharged on the 7th day after surgery.

## 3 Discussion and conclusion

There are 3 factors influence the management of pregnant women with severe pulmonary hypertension following cesarean section, including anesthesia method, vasopressors, and interspace space for epidural puncture.

General anesthesia and intraspinal anesthesia have both been reported in women with PH. During the induction period of general anesthesia, systemic vasodilation reduces blood pressure; consequently, inadequate blood return back to the right heart and decreased blood volume in the right heart will further lead to worsening of pulmonary ventilation/perfusion mismatch. Because hemodynamics fluctuates greatly during the induction period of general anesthesia and positive pressure ventilation puts pressure on the pulmonary blood vessels, and the pulmonary artery pressure may further increase, pregnant women with PH should avoid general anesthesia.^[5–7]^ Spinal anesthesia may cause significant changes in hemodynamics due to the high block level of anesthesia or the rapid rise of the block level; Sometime, the anesthesiologists are worried that the single epidural analgesia area is not enough to cover the analgesic required for the cesarean section. Therefore, continuous epidural anesthesia may be a better choice for such patients because of its slow onset and maintenance of stable blood pressure. Continuous epidural anesthesia with double catheters was initially used for obstetric labor analgesia, but it was gradually applied to postoperative analgesia, such as scoliosis surgery and esophagectomy, because it can provides better postoperative analgesia, and fewer side effects, compared with single epidural analgesia or intravenous morphine.^[8–11]^ Case reports about this anesthesia method in pregnant women with pulmonary hypertension are rare. At present, there is only 1 case report about continuous epidural anesthesia with double catheters used for cesarean section combined with mitral valve stenosis and pulmonary hypertension.^[12]^

Due to the advantages of stable hemodynamics with the help of vasopressors, dual epidural anesthesia is an option for patients with severe pulmonary hypertension and other cardiopulmonary disease. In this case, systolic blood pressure ranged from -14.4% to + 17.3%, and diastolic blood pressure decreased by 37.5% to 0.1% while adjusting the block level. Therefore, during the administration of dual epidural anesthesia, more attention should be given to changes in diastolic blood pressure, fluids can be appropriately supplemented, and vasopressors should be used. Compared with phenylephrine, norepinephrine acts on alpha and beta adrenergic receptors, so it can increase blood pressure without reducing heart rate and cardiac output.^[13]^ Infusion of 0.08 μg kg^−1^ minutes^−1^ of norepinephrine can effectively prevent hypotension in 90% of patients, with no difference in adverse events.^[14]^ The initial dose of noradrenaline infusion in our case is 0.05 μg kg^−1^ minutes^−1^, which is less than the recommended dose, that may be one of the causes of fluctuations in blood pressure.

The last factors affecting the management of pregnant women with severe pulmonary hypertension following cesarean section is the interspace space for epidural puncture. The epidural puncture gap is closely related to the level of anesthesia and the amount of local anesthetic used in the operation. Compared with single epidural anesthesia, the dual epidural approach has a more complete analgesic effect, and the amount of local anesthetic of this approach is less theoretically. Indeed, the total volume of epidural solution administered was substantial in this patient, possibly because the gap of puncture was close. The T12-L1 interspace and L3–4 interspace can be selected in the future.

After the fetus was taken out, oxytocin was pumped to contract the uterus, and the blood pressure dropped significantly to 80/55 mm Hg. After administration of oxytocin, PAP showed a general upward trend in all cases.^[5]^ Therefore, obstetricians must weigh the pros and cons before using oxytocin to avoid drastic fluctuations in hemodynamics.

In both general anesthesia and intraspinal anesthesia, the following considerations should be taken to avoid further increases in PAP: avoiding capacity overload, avoiding hypoxemia and acidosis, relieving perioperative pain, correcting anemia and improving oxygen delivery. In summary, the advantages of continuous epidural anesthesia with double catheters are stable hemodynamics and complete analgesia. This is a successful case that continuous epidural anesthesia can be safely used during cesarean section in pregnant women with pulmonary hypertension. The new knowledge of the case report is that the continuous epidural anesthesia with double catheters can be applied to patients who have a cardiopulmonary disease like severe pulmonary hypertension and do not have contraindication for epidural anesthesia and following pelvic or abdominal surgery.

## Author contributions

**Conceptualization:** Pingzhu Wang.

**Supervision:** Yushan Ma, Xiaojing Chen.

**Writing – original draft:** Pingzhu Wang.

**Writing – review & editing:** Jingwen Zhang, Yushan Ma.
